# Electronic Feedback Alone Versus Electronic Feedback Plus in-Person Debriefing for a Serious Game Designed to Teach Novice Anesthesiology Residents to Perform General Anesthesia for Cesarean Delivery: Randomized Controlled Trial

**DOI:** 10.2196/59047

**Published:** 2024-11-19

**Authors:** Allison Lee, Stephanie Goodman, Chen Miao Chen, Ruth Landau, Madhabi Chatterji

**Affiliations:** 1 Department of Anesthesiology and Critical Care, University of Pennsylvania, 3400 Spruce St, 680 Dulles, Philadelphia, PA, 19104, United States, 1 3055826077; 2 Department of Anesthesiology, Columbia University, New York, NY, United States; 3 Teachers College, Columbia University, New York, NY, United States

**Keywords:** general anesthesia, cesarean delivery, multiple choice questions, serious game, debriefing, feedback, anesthesia, anesthesiology, anesthesiologist, anesthetist, cesarean, EmergenCSim, randomized controlled trial

## Abstract

**Background:**

EmergenCSim is a novel researcher-developed serious game (SG) with an embedded scoring and feedback tool that reproduces an obstetric operating room environment. The learner must perform general anesthesia for emergent cesarean delivery for umbilical cord prolapse. The game was developed as an alternative teaching tool because of diminishing real-world exposure of anesthesiology trainees to this clinical scenario. Traditional debriefing (facilitator-guided reflection) is considered to be integral to experiential learning but requires the participation of an instructor. The optimal debriefing methods for SGs have not been well studied. Electronic feedback is commonly provided at the conclusion of SGs, so we aimed to compare the effectiveness of learning when an in-person debrief is added to electronic feedback compared with using electronic feedback alone.

**Objective:**

We hypothesized that an in-person debriefing in addition to the SG-embedded electronic feedback will provide superior learning than electronic feedback alone.

**Methods:**

Novice first-year anesthesiology residents (CA-1; n=51) (1) watched a recorded lecture on general anesthesia for emergent cesarean delivery, (2) took a 26-item multiple-choice question pretest, and (3) played EmergenCSim (maximum score of 196.5). They were randomized to either the control group that experienced the electronic feedback alone (group EF, n=26) or the intervention group that experienced the SG-embedded electronic feedback and an in-person debriefing (group IPD+EF, n=25). All participants played the SG a second time, with instructions to try to increase their score, and then they took a 26-item multiple-choice question posttest. Pre- and posttests (maximum score of 26 points each) were validated parallel forms.

**Results:**

For groups EF and IPD+EF, respectively, mean pretest scores were 18.6 (SD 2.5) and 19.4 (SD 2.3), and mean posttest scores were 22.6 (SD 2.2) and 22.1 (SD 1.6; *F*_1,49_=1.8, *P*=.19). SG scores for groups EF and IPD+EF, respectively, were—mean first play SG scores of 135 (SE 4.4) and 141 (SE 4.5), and mean second play SG scores of 163.1 (SE 2.9) and 173.3 (SE 2.9; *F*_1,49_=137.7, *P*<.001).

**Conclusions:**

Adding an in-person debriefing experience led to greater improvement in SG scores, emphasizing the learning benefits of this practice. Improved SG performance in both groups suggests that SGs have a role as independent, less resource-intensive educational tools.

## Introduction

Healthcare Simulation Standards of Best Practice dictate that a debriefing process that is grounded in theoretical frameworks or evidence-based concepts is necessary to achieve sound simulation-based experiences [1,2]. The process may use multiple techniques, including feedback, debriefing involving facilitator- or self-guided reflection, or electronic or computerized methods, and should adapt to whichever modality is being used [2,3]. Kolb [4] theorized that adult learners must undergo self-reflection before lessons may be internalized and consolidated into their existing cognitive framework. The learner may then apply the new knowledge to new situations, undergo self-reflection based on the new experience, and so on.

Serious games in health care are a type of experiential learning that have rapidly increased in popularity; however, their efficacy with respect to generating significant learning outcomes has been reportedly variable [5-10]. Experts have raised concerns that the debriefing component of games has been neglected and poorly studied [11]. Furthermore, the debriefing component, which typically uses an electronic feedback model [12], is not even consistently described in proposed design frameworks [13,14].

Electronic automated written feedback is typically provided based on the player’s expected actions being detected as “performed,” “partially performed,” or “not performed,” by the game software. Evidence of the value of electronic feedback has been previously demonstrated in 1 randomized controlled trial [15].

Notwithstanding, Cheng et al [16] stipulate that a hallmark of debriefing is the bidirectional and reflective nature of the discussion. By contrast, the feedback provided with an automated tool, although individualized, is unidirectional. Having a facilitator be a conversational guide has been considered crucial for ensuring that events that occurred during simulation-based learning are reviewed and that learning objectives are discussed [17].

The potential for learners to play serious games (SGs) independently and achieve significant learning gains using automated electronic feedback only (without a live facilitator) would amplify the flexibility and scalability of these platforms. Electronic or computerized or “self-debriefing” approaches where learners guide themselves to reflect on their performance via techniques ranging from written checklists to video tutorials have been compared with instructor-facilitated debriefing in the literature in the context of immersive full-scale scenario-based simulation [18,19], but to our knowledge, a comparison of in-person facilitator-led debriefing and electronic feedback has never been reported in the setting of SGs.

Because of the precipitous declines in trainee clinical exposure to performing general anesthesia for cesarean delivery [20], in 2016, we developed EmergenCSim, a novel researcher-developed serious 3D video game (SG) that reproduces the environment of an obstetric operating room with an embedded scoring and debriefing tool [8]. The learner, via an avatar, must perform general anesthesia for emergent cesarean delivery for the clinical scenario of umbilical cord prolapse. We hypothesized that an in-person debriefing in addition to the SG-embedded electronic feedback would provide superior learning outcomes than SG electronic feedback alone [15].

## Methods

### Research Objective

This randomized controlled trial followed a pretest-posttest design to explore the optimal debriefing style for SG-mediated instruction of CA-1 residents through a comparative evaluation of 2 models of debriefing—electronic feedback alone versus a combination of in-person debriefing and electronic feedback.

The research question examined was as follows: Is a combination of in-person and electronic feedback superior to electronic feedback alone for improving declarative and applied knowledge after playing EmergenCSim?

We hypothesized that a combination of in-person and electronic feedback would be superior to electronic feedback alone, based on an improvement in both the group’s mean SG-embedded performance score from first to second time playing the SG, and improvement in the group’s mean pretest to posttest score.

### Recruitment

Participants were clinical anesthesia year 1 (CA-1) residents from 2 consecutive classes starting their CA-1 years in 2019 and 2020 (n=51) at the Columbia University Irving Medical Center, who were randomized to 2 groups: group EF (electronic feedback only; control group; n=26) versus group IPD+EF (in-person debriefing and electronic feedback; intervention group; n=25). Noninclusion criteria included refusal to participate and prior postgraduate anesthesiology training.

In our anesthesiology residency program, 2 CA-1 residents are assigned to rotate for the first time on the labor and delivery unit, beginning in the third month of CA-1 year. Two new residents from each class continue to be assigned each subsequent month, the result being that the final 2 residents from each CA-1 class are experiencing their initial rotation by approximately the 18th month of residency (ie, 6 months into the clinical anesthesia year 2 [CA-2] year). During the week prior to the start of their initial obstetric anesthesia rotation, residents were contacted by email and informed about the study that their participation would be voluntary and declining to participate would not affect their standing in the department or the residency program.

### Instruments

Parallel, multiple-choice test forms were developed for use as pre- and posttreatment outcome measures (Multimedia Appendix 1) [21]. Test form development included (1) assessment purpose and population specification, (2) content domain specification and writing or selection of items, (3) content validation by experts (obstetric anesthesia fellowship-trained anesthesiologists with ≥10 years of clinical experience) of paired items by topic and cognitive level, and (4) empirical validation of scores from the parallel test forms using Classical Test Theory techniques [22,23]. The questions were designed to assess “higher-order thinking” that tests applied knowledge. Each item comprised a stem, 1 correct answer, and 3 distractors. The pool of questions was built upon a 26-item instrument that had been previously validated and field-tested [24]; the detailed process, which involved dropping poorly performing items from the prior instrument, revising weak but highly content-relevant items, and developing new items, has been previously published [21].

Field-testing for empirical validation involved web-based administration of 52 shuffled items from both test forms to 24 CA-1s, 21 CA-2s, 2 fellows, 1 attending anesthesiologist, and 1 of unknown rank at 3 US medical schools. Items from each form yielded near-normal score distributions, with similar medians, ranges, and standard deviations. Per Classical Test Theory, item difficulty (item *P* values) and discrimination (D) indices indicated that most items met assumptions of criterion-referenced test design, separating experienced from novice residents. Experienced residents performed better on overall domain scores than novices (*P*<.05). Kuder-Richardson Formula 20 (KR-20) reliability estimates of both test forms were above the acceptability cut of 0.70, and parallel forms reliability estimate was high at 0.86, indicating that results were consistent with theoretical expectations [22,25].

The development of the SG-embedded score was previously described in a report of a single-blinded, longitudinal randomized experiment studying the use of EmergenCSim to improve trainee knowledge regarding general anesthesia for cesarean delivery [8]. The electronic feedback script items (Multimedia Appendix 2) also used in the latter study were based on a previously validated behavioral checklist that was developed to measure resident performance of general anesthesia for cesarean delivery on a human patient simulator [26].

### Research Protocol

Three days before their initial obstetric anesthesia rotation, residents (n=25 [2019 CA-1 class], n=26 [2020 CA-1 class]) were invited by email to voluntarily participate in study activities on the third day of the rotation. They were asked to watch a 20-minute video lecture (Panopto Inc [2007], PANOPTO@COLUMBIA [version 14.0.0.00201; Carnegie Mellon University]) in advance of study participation. The lecture covered the steps for performing general anesthesia for emergency cesarean delivery and explained both the relevant underlying knowledge and the crisis resource management principles.

Individuals participated in the study activities one at a time. After verifying that the lecture had been viewed, participants provided written informed consent to participate and completed a 26-item multiple-choice question (MCQ) pretest (maximum score of 6 points, with each correct item assigned 1 point and incorrect answer assigned 0 point). Residents were then directed to watch a <3-minute video tutorial explaining how to use the game platform. The tutorial may be viewed on the web [27]. They were then invited to interact with a practice game environment using the same platform but with different avatars to familiarize themselves with how to perform actions within the game. The practice game was non–content specific and had no attached storyline.

Participants were randomized using Bernoulli randomization in R (RStudio, version 3.4.0; Posit PBC) to either the following:

1. Electronic feedback group (EF, control, n=26)
2. In-person debriefing + Electronic feedback group (IPD+EF, treatment, n=25)

**Figure 1.**
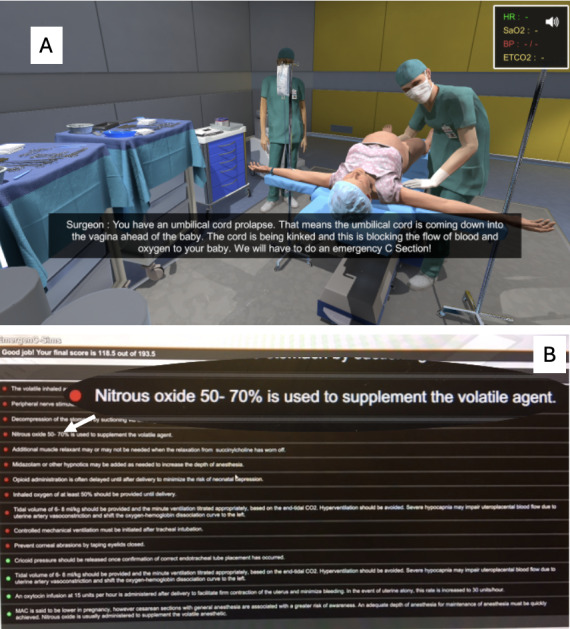
Screenshots of the serious game showing (**A**) the opening scene in the obstetric operating room where the learner encounters the obstetrician explaining to the patient that umbilical cord prolapse has occurred and (**B**) the electronic feedback screen with one of the bulleted explanations highlighted.

Before starting gameplay, they were instructed to perform actions in the game as they would in real life and informed that at the conclusion of gameplay, they would be given a score (maximum 196.5) and automated electronic feedback that would explain which actions were performed correctly or not, and why those actions were important (Figure 1B). They were not forewarned that they would be asked to play the game again.

Upon conclusion of the game and experiencing the electronic feedback (Multimedia Appendix 2), group IPD+EF received a 10-minute semistructured debriefing facilitated by AL that integrated concepts from the Promoting Excellence and Reflective Learning in Simulation (PEARLS) debriefing framework [28]. Participants were asked to reflect on the steps taken in the game and the components of management of the clinical scenario with questions such as “Can you walk me through what you were thinking when you were asked to put this patient to sleep emergently?” and “Were there any aspects of the explanations given that you did not understand or need help clarifying?” If gaps in knowledge or understanding of the concepts being taught were uncovered, directive teaching was provided. Strategies for scoring better in the game were not discussed. The control group, group EF, was exposed to the electronic feedback alone.

Regardless of group assignment, receiving feedback with or without in-person debriefing, all participants were next instructed to play EmergenCSim again, with the goal of improving their score, following which they took the MCQ posttest (maximum score of 26 points, with each correct item assigned 1 point and incorrect answer assigned 0 point). Participants were given a maximum of 26 minutes (1 minute per question) to complete each knowledge test (pretest and posttest). Following the posttest they were asked to complete a brief survey (Multimedia Appendix 3).

The survey instrument gathered demographic information, asked about prior clinical experience with performing general anesthesia for cesarean delivery or for nonobstetric surgery in pregnant patients and about the participants’ prior experience playing video games. We were also interested in gathering feedback about (1) the perception of realism of the game; (2) the level of effort required to play the game, given that cognitive load is believed to impact learning outcomes; (3) learner satisfaction with the debriefing experiences; and (4) perceptions regarding the effectiveness of the SG as a teaching tool. The survey items were written by AL and then reviewed and edited by RL and MC for clarity and meaning.

### Statistical Analysis

This was a mixed methods randomized controlled trial that obtained quantitative data to evaluate 2 models of debriefing, followed by a qualitative inquiry to explain the quantitative results. Our hypothesis was that the IPD+EF group would achieve a greater increase in written test scores (pretest to posttest) and a greater increase in SG scores (first to second gameplay) than the EF group. Participants’ reflections regarding their game playing and feedback experience, collected via the survey, explored their perceptions of the game and views regarding feedback.

### Power

The primary outcome was the difference between experimental groups in the change in mean score from pretest to posttest. Resident class sizes are fixed; however, we estimated that with an SD of 5, we would achieve 80% power to detect a 4-point difference between groups on improvement in written test scores with a significance level (α) of .05 using a 1-tailed 2-sample *t* test.

Repeated-measures ANOVA was performed for within-participant pretest-posttest scores and for between-participant variable IPD+EF and EF groups. Repeated-measures ANOVA was performed as it is the appropriate design to apply when the same group of participants is measured on 2 occasions. The repeated measurement of the same participants on the knowledge test and SG (dependent variables) caused observations in those instruments to be correlated, violating the assumptions of an independent means *t* test. The design enabled testing of the within-participant prescore-postscore change and between-participant differences with appropriate *F* tests. Repeated-measures ANOVA yields greater power to detect a true difference between groups [29]. Prior to the ANOVAs, data were checked to ensure that test assumptions had been met.

For secondary outcomes, the paired *t* test was used. Univariate analyses with the 2-sample *t* test for continuous demographic covariates and the Fisher exact test for categorical covariates were used. The correlation between group allocation and performance on the written posttest was measured by Pearson correlation coefficient. *P* value of <.05 was considered to be statistically significant. No formal qualitative analyses of the participants’ free-text responses to the perception survey were conducted. All analyses were performed using SPSS (IBM Corp Released 2021. IBM SPSS Statistics for Macintosh, version 28.0).

### Ethical Considerations

This study underwent human participants research ethics review and received the approval of the Columbia University institutional review board (AAAQ8025). The trial was not publicly registered as this was not a requirement of the review board or the funding agency for education research at the time that the trial received approval and was conducted.

Written informed consent was obtained from participants in this study and for primary data collection from participants in the prior studies from which research data were used [8,24]. Privacy and confidentiality protections that were implemented included anonymous collection of responses during empirical validation procedures of the written knowledge test outcome instrument and deidentification of the study data in the current randomized experiment. No additional consent was requested for secondary analysis of historical anonymously collected test response data. No compensation of any kind was provided for participation in research.

## Results

All 51 CA-1 residents who were invited to participate in the study provided written informed consent to participate. Demographic characteristics by study group are shown in Table 1.

**Figure 2.**
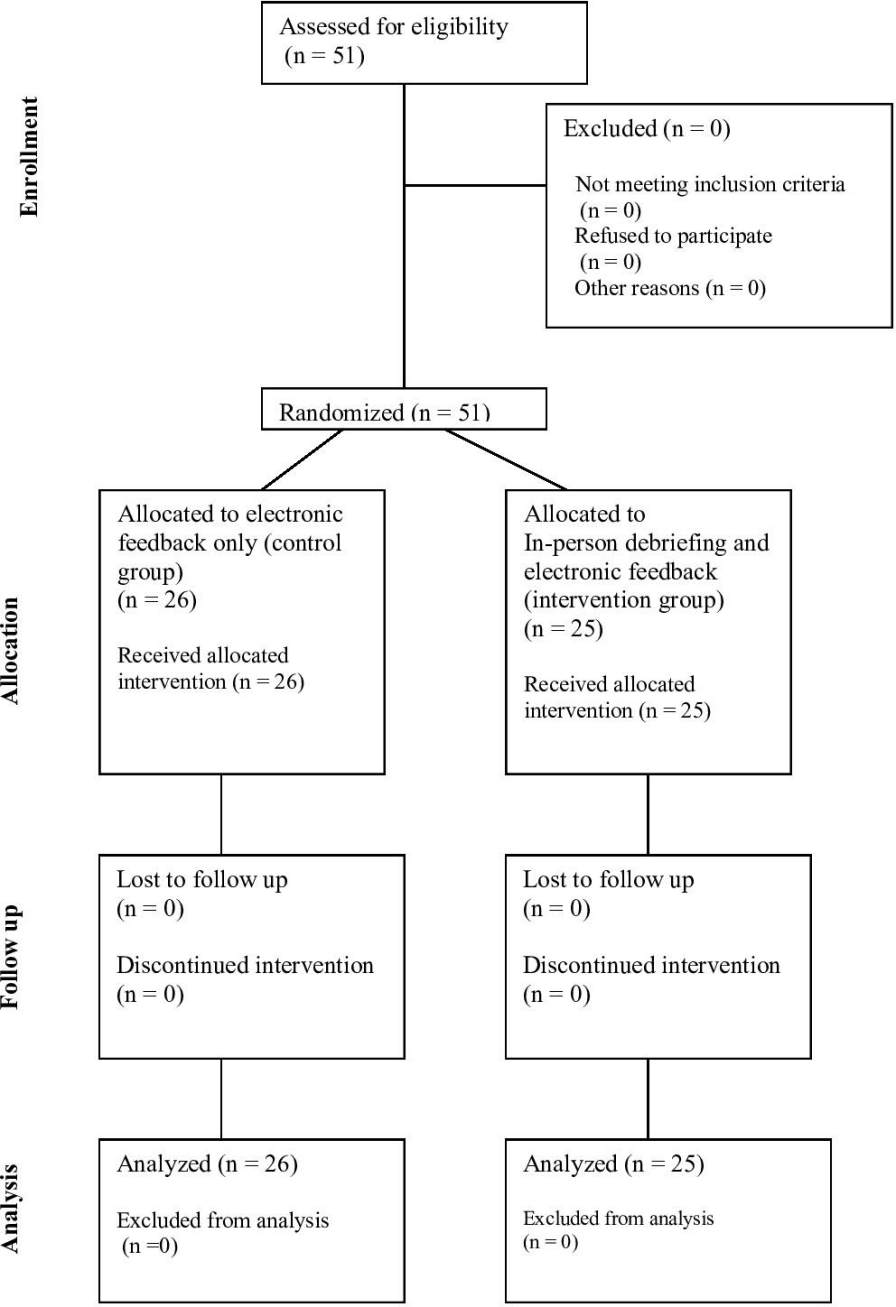
CONSORT (Consolidated Standards of Reporting Trials) diagram showing the flow of participants through each stage of a randomized controlled trial.

All participants increased their written test score from pre- to posttest (*F*_1, 49_=56.28; *P*<.01) but there was no difference between groups in the degree of improvement (*F*_1, 49_=1.8; *P*=.19; Table 2). Figure 2 presents the flow diagram of participants.

**Figure 3.**
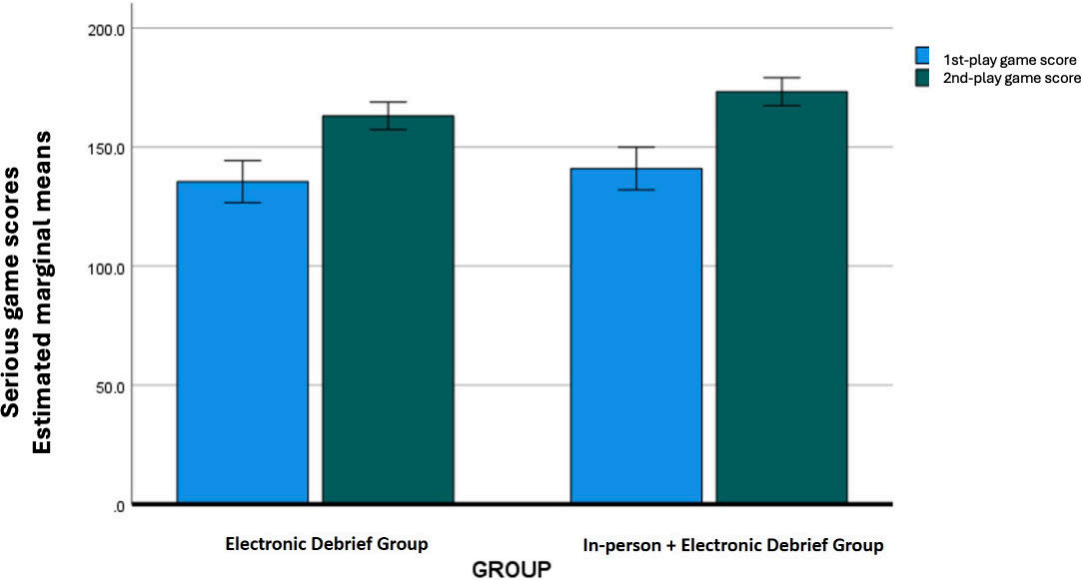
Mean game scores by experimental group; maximum 196.5 points. Error bars=95% CI.

All participants improved their SG score from the first to second gameplay; mean improvement overall 29.96 (SE 3.64; *P*<.01) points (Figure 3). There was no significant correlation between the written posttest scores and the second play game scores (*r*=0.137).

**Table 1.** Demographic characteristics of both study groups.

Study group	Electronic feedback (EF; n=26)	In-person debriefing + electronic feedback (IPD+EF; n=25)
Gender (women/men)	15/11	7/18
**Age range (years), n**		
≤25	1	0
30	20	18
35	5	5
36‐40	0	2
**Timing of participation during by clinical anesthesia year 1 or 2 (CA-1 or CA-2), n**		
1st 6 months CA-1	8	7
2nd 6 months CA-1	14	9
1st 6 months CA-2	4	9

**Table 2.** Scores on MCQ test and serious game by group^a^.

Scores	Intervention group (IPD+EF^b^; n=25)	Control group (EF^c^; n=26)	*P* value (for the score difference between groups)
**26-item MCQ^d^ scores, mean (SD)**			.19
Pretest	19.4 (SD 2.3)	18.6 (SD 2.5)	
Posttest	22.1 (SD 1.6)	22.6 (SD 2.2)
**SG^e^ scores, mean (SE)**			.02
1st (maximum: 196.5)	141.0 (SE 4.5)	135.5 (SE 4.4)	
2nd (maximum: 196.5)	173.3 (SE 2.9)	163.1 (SE 2.9)

a Data presented as mean (SD) or error (SE). Repeated-measures ANOVA was performed for within-participant and between-participant variables for the IPD+EF and EF groups with respect to the pretest-post test knowledge test and first and second SG scores.

b IPD+EF: In-person debriefing + Electronic feedback.

c EF: Electronic feedback.

d MCQ: multiple-choice question.

e SG: serious game.

After performing pairwise comparisons, participants in group IPD+EF (N=25) had significantly greater improvement in their SG performance from the first to second game play than those in group EF (N=26); mean difference between groups for second gameplay score was 10.19 (SE 4.09; *P*=.02; Table 2).

There was no statistically significant difference in performance on the SG or MCQ test based on gender.

Thirty-six participants reported having never performed general anesthesia for cesarean delivery (n=18, IPD+EF group), whereas 14 had encountered the scenario 1‐2 times (n=6, IPD+EF group), and 1 participant (IPD+EF group) had done it 3‐5 times. With respect to nonobstetric surgery in pregnancy, 23 participants had performed it 1‐2 times (n=11, IPD+EF group) and 2 participants had performed it 3‐5 times (n=1, IPD+EF). Only 8 participants reported never having played video games (n=2, IPD+EF, all were female). Twelve reported playing “very often” (more than once per month; n=6, IPD+EF, 3 were female), 3 reported playing “often” (7‐12 times per year) (n=2, IPD+EF, all were male), and 14 “occasionally” (1‐12 times per year; n=10, IPD+EF).

### Perceptions of EmergenCSim

The overall mean rating of game realism (scale 1‐5, where 1=not realistic at all, 5=very realistic) was 3.78 (SD 0.673). Participants (n=51) gave a mean rating of the level of mental effort required to play the SG (scale 1=very very low mental effort, 9=very very high mental effort) of 6.43 (SD 1.42). Reasons given for the answers related to lack of intuitiveness of use of the game, including finding it cumbersome to use multiple clicks to perform actions, and difficulty with certain aspects of the game, especially with respect to providing oxygen to the patient avatar. The full list of free-text responses regarding participant perceptions of EmergenCSim is shown in Multimedia Appendix 4.

With respect to their reported level of stress playing the game (responses were scored on a 5-point scale ranging from “not stressed at all” to “very stressed”), 28 felt quite or somewhat stressed, 21 felt slightly stressed, and 2 felt not stressed at all. The stress was reported to be related to constant questions from the patient (eg, “How is my baby doing?”) and the surgical team (eg, “Is she anesthetized?”) and the sense of time pressure for the scenario. One person reported feeling low stress because “the repercussions for mistakes were low.” The full set of free-text responses regarding the level of stress felt while playing the game is shared in Multimedia Appendix 4.

### Satisfaction With Debriefing

Participants who received in-person debriefing after playing the SG (n=25) rated their satisfaction with this type of debriefing (scale 1‐5, where 1=not at all satisfied, and 5=very satisfied) as either 4 (n=5) or 5 (n=18), mean of 4.78 (SD 0.42). The resident rating of the electronic feedback (n=51) was slightly lower (mean 4.22, SD 0.80).

### Perception of the Usefulness of SGs for Teaching

Regarding the question “Knowledge gained from playing a serious game can be transferred to the clinical setting” (scale 1‐5, where 1=strongly disagree, 5=strongly agree), most participants either agreed (n=21) or strongly agreed (n=28) and 2 were neutral (neither agree nor disagree). New information learned from playing EmergenCSim primarily centered on the use of nonparticulate antacids for gastrointestinal prophylaxis, how to use nitrous oxide to limit the concentration of volatile anesthetic agents administered, delaying administration of intravenous opioids and supplemental hypnotic agents until after delivery of the neonate, and crisis management principles such as calling for help early. The full list of free-text responses is listed in Multimedia Appendix 4.

## Discussion

### Principal Findings

We found that among novice anesthesiology residents who played an SG of a scenario involving the performance of general anesthesia for emergency cesarean delivery, an in-person facilitated debriefing in addition to the game-embedded electronic feedback after initial gameplay resulted in significantly higher improvement in game performance scores on the second play, compared with the control group that received only the electronic feedback. To our knowledge, this is the first study to compare learning outcomes associated with electronic feedback alone compared with a combination of electronic feedback and in-person debriefing for an SG.

Our study takes the learner through the phases of Kolb’s cycle of experiential learning, starting with the concrete experience of playing the SG, then the reflective observation and abstract conceptualization provided via the feedback and debriefing steps, followed by the active experimentation of applying what was learned, with the opportunity to replay the game [4,30].

Electronic or computerized feedback tools most closely resemble self-directed debriefing approaches successfully described with immersive full-scale scenario-based simulation—these may be either video-assisted or conducted with the use of cognitive aids and have been associated with similar learning outcomes compared with instructor-led debriefing [31,32]. These findings also align with adult learning theory since adult learners are believed to be intrinsically motivated, prefer autonomy and being responsible for their own learning, and learn better with problem-focused content [33]. The unidirectional nature of electronic feedback differs from the traditional, bidirectional debriefing approaches [2]; advances in artificial intelligence technology in the future may facilitate bidirectional feedback via the technological platform [34].

We speculate that greater psychological safety may be attained during self-directed learning [35]. As mentioned earlier, computerized feedback has been demonstrated to be superior to no debriefing at all and not all games are explicitly designed with an embedded feedback tool [15]. The ability to produce knowledge gains without a human instructor boosts the cost-effectiveness, flexibility of independent learner access, and use of this learning modality [36].

Traditional “terminal debriefing,” at the end of an event, is an interactive, instructor-led discussion, aimed at leading guided reflection for the learner, with the goal of closing knowledge and skill gaps [16,17], and debriefing with even as short a duration as our in-person component has been shown to be effective in enhancing knowledge gains [37]. A study comparing facilitated debriefing, feedback, and self-debriefing for human patient simulations found greater improvement in scores with facilitated debriefing and that both students and faculty valued facilitated debriefing over the other 2 modalities [38]. A study exploring nursing student perceptions of self-debriefing which occurred in advance of a facilitated group debriefing found that self-debriefing increased learners’ self-awareness and ability to reflect on knowledge gaps and make connections to clinical practice; however, an extended richer reflection occurred in the context of the group debriefing, supporting the value of a combination of approaches [18]. Among our residents, their reported satisfaction with in-person debriefing and electronic feedback was only slightly greater for in-person (4.78) versus electronic (4.22), with no statistically significant difference, which suggests that they considered electronic feedback to be acceptable and effective. This perception could have been influenced by the specific study context of screen-based simulation—preferences and expectations might have been different had this been an immersive full-scale scenario–based simulation.

### Comparison to Prior Work

Midwifery students (n=28) participating in screen-based simulation training on neonatal resuscitation, who were randomized to receive what the authors termed, “computer debriefing” versus “no debriefing,” demonstrated greater improvement in nontechnical skills (anesthetists’ nontechnical skills [39] system score of 13.25 vs 9; *U*=47.5; *P*=.02); they also scored higher on self-efficacy using a 6-point Likert scale, 0=“not at all confident” to 5=very confident” (3 vs 2; *U*=52; *P*=.02), and had greater improvement in knowledge (a baseline difference of 13 in the debriefing group vs 14.5 for control group was eliminated; *P*=.05) [15].

Our findings also suggest that SGs that provide embedded electronic feedback may be effective for learning the applied knowledge required to perform complex clinical scenarios; the provision of in-person, facilitated debriefing further amplifies learning gains, likely due to the bidirectional, interactive nature. Correspondingly, Dreifuerst et al [40] have promoted use of the “debriefing with meaningful learning” approach for screen-based simulation. The technique uses reflection-in-action, reflection-on-action, and reflection-beyond-action to teach clinical reasoning. Learners document and reflect on their actions using worksheets while the debriefer is reviewing the computer-generated performance reports. Videoconferencing platforms then allow learners and facilitators (in remote locations) to have an interactive group discussion of the key issues to uncover the learners’ thinking and assumptions.

One systematic review reported that among 11 experimental studies assessing participants’ acquisition of knowledge as a result of playing SGs, a negligible and nonstatistically significant standardized mean difference was found in favor of SGs, although interestingly, subgroup analyses found a significant difference among studies involving health care students as opposed to health care professionals [5]. Learning outcomes with computerized, screen-based simulators such as SGs appear to be maximized when learners are able to interact with the interfaces repeatedly [7,40]. The opportunity to replay the scenario may be appealing to learners who are motivated to perfect their performance [41]. The drawback of a fixed scenario may be the lack of variability that is normally encountered in clinical practice, giving learners, who achieve high scores, a false sense of security regarding their skills and knowledge [40].

All participants increased their written test score from pre- to posttest. Although there was no statistically significant difference between groups, the improvement in score on the parallel test forms indicates that learning did occur in both groups, although a difference could not be detected by treatment group. The lack of a difference in improvement between groups on pre- to posttest scores highlights the difficulty of assessing knowledge gains for a complex clinical scenario that covers multiple domains.

Most residents reported that they were “quite” or “somewhat stressed” during gameplay. In real clinical practice, the conduct of general anesthesia for emergency cesarean delivery is extremely stressful, with pressure placed on the anesthesia providers to anesthetize the patient as quickly and as safely as possible. Repeated questions by the avatar representing the obstetric surgeon were intentional to mimic the real context. The mean rating of 3.78 for realism of the game (scale 1‐5) was moderately realistic, and the level of mental effort required to play the game was given a mean rating of 6.43 (scale 1‐9). Future studies and iterations of the game should aim to reduce cognitive load further, while enhancing the immersive feel and realism for learners.

Long-term memory is believed to be the dominant structure from which learners draw during problem-solving, whereas conscious processing is thought to occur using working memory, which is limited in its duration and capacity [42]. The relevance to SG design is that if working memory is overloaded during the exploration of a complex new environment, learning may be diminished [43]. Novice learners, who lack the underlying schema to integrate the new information, may be more negatively impacted by unguided tools. Our goal with game design was to minimize *extraneous cognitive load* (the working memory resources for task completion that do not enhance learning) and maximize *germane load*, a subtype of intrinsic load that engages learners and leads them to the construction of desired schemas in long-term memory [43].

Experiential learning involves active participation and often triggering of intense emotions, which are both believed to promote long-lasting learning effects [44]. It was gratifying to see that virtually all the residents found the experience of playing the game beneficial and were able to report specific areas of knowledge gained.

### Limitations

The primary limitations of this study are first the small sample size due to the typically small resident class sizes and second, the difficulty in achieving clean experimental conditions between treatment and control groups.

Larger sample sizes could be achieved by involving participants at the identical level of training from multiple similar academic centers; however, a large number of disparate centers would threaten the internal validity of the study by introducing heterogeneity with respect to the learning environment and backgrounds of learners. It is possible that residents discussed the study with their classmates and conducted varying levels of advance preparation for the rotation.

Third, residents unavoidably experienced their initial obstetric anesthesia rotation at different times during their first 18 months of residency, so there was heterogeneity in their overall level of clinical experience. All residents participated during their initial obstetric anesthesia rotation when they were assumed to be unfamiliar with the scenario being taught and when the relevance of the content might produce high motivation for learning. Randomization to experimental groups was performed at the beginning of the CA-1 academic year and the timing of the initial obstetric anesthesia rotation for each resident was determined by the residency program. Several residents reported having had some prior experience with the scenario or else managing anesthesia for nonobstetric surgery in pregnant patients, where some of the anesthetic implications are similar to that for cesarean delivery. We were not able to intentionally equalize the level of clinical experience between groups. We think that this is not likely to have significantly impacted the study outcomes; all were on their first-ever obstetric anesthesia rotation and there was not a large degree of imbalance between groups according to level of clinical experience.

Fourth, the intervention group, by virtue of the time spent on debriefing, spent more time reflecting on the SG. It is possible that the longer time spent in reflection was the cause of the greater improvement in test scores. It is unclear whether, if given time to reflect on the game, as opposed to engaging in debriefing, a similar improvement in SG scores would have occurred.

### Future Directions

Future research should focus on the optimization of the game platform with respect to usability and on iteratively making improvements based on the feedback of players. Continued research into the best practices for debriefing for SGs, timing, variations in structure and need for in-person versus web-based facilitation, ways to incorporate group debriefing, and the role of using artificial intelligence [34,45] is warranted to maximize the learning benefit from these teaching tools. Rigorous validation of the assessment tools for the measurement of learning gains is crucial. Finally, discovering ways to link the learning gains with these educational tools to real-world clinical performance and outcomes would be highly desirable for establishing their use in health care education, including studies of ultimate cost-benefit ratio [10,46].

### Conclusions

The dramatic decline in the use of general anesthesia for cesarean delivery in recent decades has resulted in decreased exposure of anesthesia residents to the management of this scenario, leading to significant interest in developing innovative alternative strategies for teaching [47]. We have shown that regardless of debriefing approach, there was improvement in learners’ cognitive and applied knowledge in the domains being taught, based on improvement in their written test and SG scores. Our findings indicate that SGs have the potential to be used independently as educational tools. The greater improvement in game performance in the group that received an in-person debriefing indicates that individualized, in-person debriefing further strengthens the learning benefit from using SGs among trainees in graduate medical education.

## Supplementary material

10.2196/59047 Multimedia Appendix 1 Pretest and posttest multiple choice questions.

10.2196/59047 Multimedia Appendix 2 Electronic feedback script.

10.2196/59047 Multimedia Appendix 3 Survey questionnaire.

10.2196/59047 Multimedia Appendix 4 Survey free-text responses.

10.2196/59047 Checklist 1 CONSORT-EHEALTH (Consolidated Standards of Reporting Trials of Electronic and Mobile Health Applications and Online Telehealth) checklist.

## Abbreviations

CA-1: clinical anesthesia year 1
CA-2: clinical anesthesia year 2
EF: electronic feedback
IPD: in-person debriefing
KR-20: Kuder-Richardson Formula 20
MCQ: multiple-choice question
PEARLS: Promoting Excellence and Reflective Learning in Simulation
SG: serious game
