# Supplementary material for: Electronic Feedback Alone Versus Electronic Feedback Plus in-Person Debriefing for a Serious Game Designed to Teach Novice Anesthesiology Residents to Perform General Anesthesia for Cesarean Delivery: Randomized Controlled Trial
Source: JMIR Serious Games. 2024 Nov 19;12:e59047. doi: 10.2196/59047 (PMC11611795; doi:10.2196/59047)
Supplement: Multimedia Appendix 1 [file games-v12-e59047-s001.pdf]

PRE-TEST

Welcome!

Thank you for agreeing to participate in this education research activity! We hope you will find this both educational and fun. In advance of the other activities, please complete this brief set of 26 MCQ's.

Each question has FOUR answer choices. Read the question and answer choices *carefully* and select the **ONE** best answer. Please answer **all** questions, even the ones you may not be sure about. There is <u>no penalty</u> for guessing!

Your answers will not be reported to your supervisors, and will not affect your standing in your department in any way. This should take approximately 10 - 12 minutes to complete.

Sincerely,

Allison Lee, MD, MS
Columbia University, New York, NY

*This work is supported by a Foundation for Anesthesia Education and Research (FAER) Research in Education Grant.

Q1 *

Which of the following muscle relaxant regimens is MOST appropriate for a fasted healthy term pregnant woman undergoing intravenous (IV) induction of general anesthesia?

- Rocuronium 0.6 mg/kg IV
- Rocuronium 1.2 mg/kg IV
- Succinylcholine 1 mg/kg IV
- Succinylcholine 1.5 mg/kg IV

Q2 *

In a laboring term patient, which of the following fetal heart rate ranges is MOST non-reassuring?

- Recurrent early decelerations that nadir at 65 bpm
- Recurrent late decelerations that nadir at 110 bpm
- Episodic variable decelerations that nadir at 65 bpm
- Persistent fetal heart rate of 170's bpm

Q3 *

Which of the following statements describes the CORRECT sequence of steps for rapid sequence induction of general anesthesia?

- Apply cricoid pressure at 30 newtons, administer induction agents, decrease cricoid pressure to 10 newtons and perform direct laryngoscopy
- Apply cricoid pressure at 10 newtons, administer induction agents, increase cricoid pressure to 30 newtons and perform direct laryngoscopy
- Administer induction agents, apply cricoid pressure 10 newtons, perform direct laryngoscopy and increase cricoid pressure to 30 newtons during laryngoscopy
- Administer induction agents, perform direct laryngoscopy and apply cricoid pressure 10 newtons during laryngoscopy

Q4 *

During general anesthesia for cesarean delivery, BEFORE delivery of the fetus, what end-tidal concentration of inhaled volatile agent is MOST appropriate?

- > 1 MAC
- 1 MAC
- < 1 MAC
- Avoid inhaled agents before delivery

Q5 *

A 36 year old term G2 P1 is undergoing cesarean delivery with general anesthesia. She is receiving 2% sevoflurane (end-tidal) and 50% nitrous oxide. AFTER delivery of the neonate, which of the following changes should be made to the inhaled agent concentrations?

- Decrease the sevoflurane level and decrease the nitrous oxide level
- Increase the sevoflurane level and decrease the nitrous oxide level
- Maintain the sevoflurane and nitrous oxide levels
- Decrease the sevoflurane level and increase the nitrous oxide level

Q6 *

In an emergency cesarean delivery with general anesthesia, which of the folllowing time points is the MOST appropriate to tell the obstetricians to make skin incision?

- At induction of anesthesia
- At visualization of the larynx
- At appearance of the continuous end-tidal carbon dioxide waveform
- At initiation of mechanical ventilation

Q7 *

During cardiopulmonary resuscitation of a term pregnant woman, which maneuver is MOST indicated for relieving inferior vena cava compression by the gravid uterus ?

- 30 degree left tilt of the surgical table
- 15 degree wedge placed under the left hip
- Supine position with left manual uterine displacement
- Trendelenburg position

Q8 *

A term parturient has INCREASED sensitivity to which of the following types of medications?

- Nonsteroidal anti-inflammatory drugs
- Volatile anesthetic agents
- Non-depolarizing neuromuscular blocking agents
- Sympathomimetic agents

Q9 *

What is the normal maternal arterial partial pressure of carbon dioxide ($PaCO_2$) at term?

- 42 - 46 mmHg
- 35 - 39 mmHg
- 28 - 32 mmHg
- 21 - 25 mmHg

Q10 *

Which of the following actions typically takes place BEFORE rapid sequence induction (RSI) of general anesthesia for elective cesarean delivery?

- Abdominal preparation and draping
- Initiation of phenylephrine infusion
- Administration of intravenous midazolam
- Placement of orogastric tube

Q11 *

You are inducing general anesthesia for emergent cesarean delivery in a term parturient. Shortly after administering the induction agents (prior to intubation of the trachea) you observe rapid oxygen desaturation from 99% to 95%. What is the MOST likely cause?

- Decreased vital capacity and increased intrapulmonary shunting
- Decreased functional residual capacity and increased oxygen consumption
- Increased minute ventilation and decreased tidal volume
- Increased closing capacity and decreased oxygen consumption

Q12 *

During an episode of prolonged fetal bradycardia intrapartum, what MINIMUM concentration of oxygen (FiO_2) will significantly increase the oxygen saturation of the fetus?

- 0.30
- 0.40
- 0.50
- 0.60

Q13 *

When initiating oxytocin administration in a term gestation patient undergoing emergency cesarean delivery, which of the following regimens is MOST appropriate?

- Intravenous infusion at 15 units/hour at skin incision
- Intramuscular injection 10 units upon uterine incision
- Intravenous push 3 units upon delivery of the neonate
- Intramuscular injection 5 units upon delivery of the placenta

Q14 *

What intravenous (IV) dose of fentanyl is usually administered during induction of general anesthesia for cesarean delivery in a heathy parturient?

- 0 mcg/kg
- 0.5 mcg/kg
- 1 mcg/kg
- 1.5 mcg/kg

Q15 *

After delivery of the neonate, which of the following conditions is the MOST comon cause of postpartum hemorrhage?

- Placenta accreta spectrum disorder
- Genital tract trauma
- Placental abruption
- Uterine atony

Q16 *

A patient is post-ictal but responsive to commands following an eclamptic seizure in the emergency room. The obstetrician would like you to proceed with general anesthesia for emergent cesarean delivery. Which are the MOST important aspects of a focused history and physical exam?

- Time of last meal, airway examination and current weight
- Medication allergies, airway examination and blood pressure
- Time of last meal, airway examination and blood pressure
- Medication allergies, current weight and blood pressure

Q17 *

In a parturient with severe gastroesophageal reflux disease who must undergo cesarean delivery with general anesthesia, which two medications are MOST indicated for decreasing the risk of chemical pneumonitis?

- Sodium citrate and ranitidine
- Sodium citrate and metoclopramide
- Ondansetron and metoclopramide
- Ranitidine and ondansetron

Q18 *

You are interviewing an 18-year-old preeclamptic G1 P0 at 28 weeks' gestation receiving a magnesium sulfate infusion.The patient has a generalized tonic-clonic seizure. In addition to evaluating her airway, breathing and circulation, which of the following are the MOST appropriate next steps?

- Administer phenytoin and notify the obstetrician
- Administer propofol and secure the airway
- Call for a code blue and administer lorazepam IV
- Notify your attending and administer a magnesium bolus IV

Q19 *

A patient with a Category 3 fetal heart rate tracing is unexpectedly rushed into your operating room for stat cesarean delivery. You did not have time to do a thorough set up of your anesthesia machine. Which of the following aspects of the machine function is MOST appropriate to check?

- Oxygen flush valve
- Oxygen analyzer
- Oxygen cylinder pressure
- Oxygen flowmeters

Q20 *

A healthy fasted 34-week pregnant woman requires general anesthesia for fetal surgery. Which of the following approaches to intravenous induction of anesthesia is MOST appropriate?

- Slow, controlled induction with midazolam, propofol and rocuronium
- Slow controlled induction with lidocaine, etomidate and succinylcholine
- Rapid sequence induction with lidocaine, propofol and succinylcholine
- Rapid sequence induction with fentanyl, ketamine and rocuronium

Q21 *

During cesaren delivery under general anesthesia, the obstetrician reports significant uterine atony. Administration of which of the following anesthetic agents is MOST likely contributing to this effect?

- Sevoflurane 1%
- Desflurane 6%
- Propofol 100 mcg/kg/min
- Ketamine 2.5 mg/kg/hr

Q22 *

During cesarean delivery, the obstetrician requests that the oxytocin intravenous infusion set be taken off the infusion pump and asks for the drip to be delivered "wide open". What would be your primary concern with complying with that request?

- Bronchoconstriction
- Uterine relaxation
- Hypotension
- Headache

Q23 *

Before general anesthesia for emergent cesarean delivery which of the following are MOST important to assess?

- Bilateral breath sounds, oxygen saturation and heart rate
- Body mass index, blood pressure and oxygen saturation
- Blood pressure, heart rate and oxygen saturation
- Blood pressure, oxygen saturation and Mallampati score

Q24 *

Which of the following perioperative complications is MORE common in a term pregnant woman vs. a non-pregnant woman undergoing general anesthesia?

- Delayed emergence
- Failed intubation
- Post-operative nausea and vomiting
- Chronic post-operative pain

Q25 *

A decrease in which of the following factors MOST contributes to a laboring parturient's risk of aspiration under general anesthesia?

- Gastric volume and lower esophageal sphincter tone
- Gastric emptying and gastric pH
- Gastric pressure and gastric pH
- Gastric pressure and gastric emptying

Q26 *

Which pregnancy-related change MOST contributes to increasing the risk of difficult intubation at term?

- Increase in breast size
- Increase in oxygen consumption
- Decrease in lumbar lordosis
- Decrease in functional residual capacity

POST-Test

Welcome!

You're on the home stretch! We hope you have been enjoying this so far....We will now ask you to complete the 26 -item MCQ post-test.

Again, each question has FOUR answer choices. Read the question and answer choices *carefully* and select the **ONE** best answer. Please answer **all** questions, even the ones you may not be sure about. There is no penalty for guessing!

After the MCQ's, there is a brief survey at the end so we can get to understand more about your experiences and perceptions. This should take < 15 minutes to complete.

Sincerely,

Allison Lee, MD, MS
Columbia University, New York, NY

*This work is supported by a Foundation for Anesthesia Education and Research (FAER) Research in Education Grant.

Multiple Choice Questions

Q1*

Which of the following airway features BEST explains why the tracheas of pregnant patients are more difficult to intubate than non-pregnant?

- Increased neck circumference
- Prominent incisors
- Increased upper airway edema
- Shorter thyromental distance

Q2*

In a pregnant woman who is not in labor, which of the following factors MOST contributes to gastro-esophageal reflux symptoms?

- Decreased gastric emptying and gastric pH
- Decreased lower esophageal sphincter tone and gastric pH
- Increased lower esophageal spincter tone and gastric volume
- Increased gastric volume and gastric pressure

Q3*

With respect to intubating the tracheas of pregnant women vs. non-pregnant women which statement is true?

- Rates of difficult/failed intubation in pregnancy have significantly decreased in the last 20 years
- Pulmonary aspiration is the primary cause of maternal deaths associated with tracheal intubation
- The risk of failed intubation is eight times higher in pregnancy
- Failed intubation in pregnancy is associated with comparable morbidity to non-pregnant women

Q4*

For emergency cesarean delivery, which step during patient preparation and assessment is MOST acceptable to skip?

- Pulse oximetry monitoring
- Electrocardiogram monitoring
- Airway exam
- Allergy history

Q5*

On which system does oxytocin produce its MOST common dose-dependent side effects?

- Central nervous system
- Cardiovascular system
- Respiratory system
- Gastrointestinal system

Q6*

A previously laboring parturient who is receiving inhaled general anesthesia is experiencing excessive bleeding after delivery of the neonate at cesarean delivery. The obstetrician reports significant atony of the lower uterine segment. Which change in management is the BEST step to reduce bleeding risk?

- Increase the intravenous lactated Ringer's infusion rate from 3 ml/kg/hr to 8ml/kg/hr
- Increase the intravenous oxytocin infusion rate from 30 units/hour to 80 units/hour
- Decrease the inhaled nitrous oxide concentration from 70% to 50% and increase the sevoflurane concentration from 1% to 2%
- Decrease the inhaled sevoflurane concentration from 2% to 1% and add a propofol intravenous infusion at 40 mcg/kg/min

Q7*

You are conducting intravenous (IV) induction of general anesthesia for elective repeat cesarean delivery of a fasted term pregnant woman with Harrington rods. Which steps are MOST appropriate before tracheal intubation?

- Push IV propofol and rocuronium, then perform bag/mask ventilation until the loss of train-of-four twitches
- Push IV propofol and succinylcholine, then perform bag/mask ventilation until after the patient has exhibited fasciculations
- Apply cricoid pressure, then push IV fentanyl, propofol and rocuronium (no bag/mask ventilation)
- Apply cricoid pressure, then push IV lidocaine, propofol and succinylcholine (no bag/mask ventilation)

Q8*

In an emergency, which equipment re-check is MOST important to perform immediately prior to induction of general anesthesia?

- Fill level of the vaporizer agent
- Functioning suction canister, tubing and cannula
- Fresh carbon dioxide absorbent
- Calibration of the oxygen analyzer

Q9*

A 38-year-old G4 P2 at 34 weeks' gestation is being transferred directly to the operating room with severe hemorrhagic shock due to suspected placental abruption. You are the first anesthesia team member to arrive. When is it MOST appropriate to call your attending to the room?

- Upon performing a focused history and examination of the patient
- Upon being notified about the case
- Upon beginning pre-oxygenation
- Upon application of monitors

Q10*

Regarding oral sodium citrate admnistration prior to general anesthesia in a term pregnant woman, which of the following statements is true?

- It should be administered at least 1 hour prior to induction
- It should be administered immediately prior to induction
- Gastric volume rapidly decreases
- Gastric pH rapidly decreases to < 2.5

Q11*

A term patient with brisk vaginal bleeding and is brought emergently to the operating room for repeat cesarean delivery. Immediately prior to inducing general anesthesia which questions are the MOST important to ask her now?

- Current medical problems, past anesthetic history and prior blood transfusions
- Current medical problems, medication allergies and family history of malignant hyperthermia
- Past anesthetic history, current medications and time of last meal
- Time of last meal, current medications and family history of malignant hyperthermia

Q12*

After delivery of the neonate, which of the following is the primary mechanism of hemostasis?

- Vasoconstriction
- Clamping of the umbilical cord
- Primary clot formation
- Myometrial contraction

Q13*

What is the usual timing of initial opioid administration during general anesthesia for cesarean delivery in a healthy parturient?

- At induction
- Immediately prior to incision
- Immediately after delivery of the neonate
- At skin closure

Q14*

For a patient experiencing postpartum hemorrhage during cesarean delivery, which of the following intravenous infusion dose regimens for oxytocin is MOST appropriate?

- 5 milliunits/minute
- 10 units/hour
- 15 milliunits/minute
- 20 units/hour

Q15*

During maintenance of general anesthesia for cesarean delivery in a healthy term pregnant woman with non-reassuring fetal status, which inhaled gas mixture with oxygen below, is MOST appropriate BEFORE delivery?

- Air 5L/min: Oxygen 1L/min
- Air 4L/min: Oxygen 2L/min
- Nitrous oxide 3L/min: oxygen 3L/min
- Nitrous oxide 4L/min: oxygen 2L/min

Q16*

A few seconds after intubation of the trachea of a term parturient for elective cesarean delivery, the maternal oxygen saturation is noted to be 93%. Which of the following is the MOST likely reason?

- Right bronchial (“main stem”) intubation
- Pulmonary aspiration of gastric contents
- Inadequate pre-oxygenation
- Increased oxygen consumption

Q17*

Which of the following steps is LEAST important BEFORE induction of general anesthesia (RSI) for emergent cesarean delivery?

- Abdominal preparation and draping
- Pre-oxygenation
- Pre-medication with midazolam
- Gastrointestinal prophylaxis

Q18*

You are managing a term pregnant woman undergoing emergency cesarean delivery with general anesthesia. Her end-tidal CO_2 concentration is 36 mmHg. Which of the following states is represented by this value?

- Hypercarbia
- Normocarbia
- Hypocarbia
- Need more information

Q19*

A term parturient has DECREASED sensitivity to which of the following types of medications?

- Nonsteroidal anti-inflammatory drugs
- Volatile anesthetic agents
- Depolarizing neuromuscular blocking agents
- Sympathomimetic agents

Q20*

Immediately after induction of general anesthesia in a parturient with a twin pregnancy at 36 weeks' gestation, her systolic blood pressure falls from 110 mmHg to 70 mmHg. In addition to fluids and vasopressors, what positioning manuever would be MOST effective to improve maternal hemodynamics before delivery?

- Bilateral passive leg raise
- Trendelenburg position
- Supine position with right manual uterine displacement
- 15 degree left tilt of the surgical table

Q21*

Clear communication with the surgical team during induction of general anesthesia for emergency cesarean delivery is MOST important to avoid which of the following issues?

- Delayed antibiotic administration
- Failure to perform surgical time out
- Foley catheter insertion before induction of anesthesia
- Premature skin incision

Q22*

For a healthy patient undergoing general anesthesia for cesarean section, which inhaled agent end-tidal concentration is MOST appropriate for maintenance of anesthesia AFTER delivery ?

- 1% sevoflurane and 70% nitrous oxide
- 1% sevoflurane and 50% nitrous oxide
- 2% sevoflurane, 50% oxygen with air
- 1% sevoflurane with 70% oxygen with air

Q23*

Following induction of general anesthesia for emergent cesarean delivery in a healthy parturient with nonreasuring fetal heart rate, what end-tidal concentrations of inhaled agents are MOST appropriate BEFORE delivery of the neonate? (Assume no intravenous maintenance anesthetics are being concurrently administered).

- 0.5% sevoflurane level and 50% nitrous oxide
- 3% sevoflurane and 70% nitrous oxide
- 0.5% sevoflurane and 70% nitrous oxide
- 2% sevoflurane and 50% nitrous oxide

Q24*

What is the correct time to begin application of cricoid pressure during rapid sequence induction?

- Immediately before administration of induction drugs
- Immediately after administration of induction drugs
- When the eyelash reflex is abolished
- When the patient becomes apneic

Q25*

During labor, which fetal heart rate range in the term fetus is MOST reassuring?

- 150 - 190 bpm
- 130 - 170 bpm
- 120 - 160 bpm
- 100 - 140 bpm

Q26*

Compared to dosing in a non-pregnant woman, which statement BEST describes the appropriate induction dose of succinylcholine in pregnancy?

- Decreased dose of succinylcholine
- Unchanged dose of succinylcholine
- Increased dose of succinylcholine
- Succinylcholine should be avoided in pregnancy
