# Supplementary material for: Electronic Feedback Alone Versus Electronic Feedback Plus in-Person Debriefing for a Serious Game Designed to Teach Novice Anesthesiology Residents to Perform General Anesthesia for Cesarean Delivery: Randomized Controlled Trial
Source: JMIR Serious Games. 2024 Nov 19;12:e59047. doi: 10.2196/59047 (PMC11611795; doi:10.2196/59047)
Supplement: Multimedia Appendix 2 [file games-v12-e59047-s002.pdf]

EmergenCSim™ Electronic Feedback Script

Action Tracked in the Game	Indicator next to each item	Debriefing Comment
**Preoperative assessment**	Correctly performed = ✅<br>Incorrectly performed = ⛔	
Introduce yourself		This may be an emergency, but always remember to do quick introductions. Greet the patient, tell her your name and explain who you are.
Obtain pertinent obstetric history		Find out the patient's gravidity, parity, gestational age, information about previous cesarean sections, previous peripartum complications, and current obstetric problems, such as preeclampsia.
Medical history		A quick systems review can be obtained with the simple question, "Do you have any medical problems?" Find out about significant medical issues with a focus on cardiopulmonary medical history. With limited time, some of this information can be obtained from the obstetricians and nurses during hand-off.
Medications		You need to know what medications she is taking in case there are implications for your anesthetic management.
Allergies		One of the most important pieces of the medical history is the allergy history. Being aware of any drug and/or latex allergies is critical to avoiding life-threatening anaphylaxis.

Previous anesthetic/family anesthetic history		Any past anesthesia complications, such as a past history of a difficult intubation helps you to make appropriate preparations. Quickly ask about any personal/family history that could suggest an episode of malignant hyperthermia.
Airway examination		Airway examination is probably the most important part of the physical examination for an anesthesiologist to anticipate the difficulty of intubation.
Call for help		In an emergency an assistant can help you complete multiple time-sensitive critical tasks, and help with clinical problem solving. At your level of training, this should be the attending anesthesiologist.
**Preoperative patient care**		
Administer 100% oxygen by mask		Pregnant women desaturate quickly because of decreased functional residual capacity and greater metabolic demands. In an emergency, adequate maternal preoxygenation may be achieved by 8 – 10 vital capacity breaths.
Left uterine displacement		Tilting the operating table 15 degrees to the left has been traditionally believed to help relieve aortocaval compression by the gravid uterus.
Ensure working intravenous catheter		Intravenous access is important for the administration of fluids and drugs. At least an 18G IV is

		preferred in the obstetric patient.
Apply blood pressure cuff		Maternal blood pressure must be measured at baseline and then at least every 5 minutes throughout the case. Around the time of induction, more frequent measurements, such as every 1 minute, are advisable.
Apply pulse oximeter		Pulse oximetry is the standard method of assessing oxygenation and must be employed during all anesthetics.
Apply electrocardiogram leads		A continuous electrocardiogram should be displayed throughout the case.
**Equipment availability check**		
Quick circuit check		Before induction, ensure that the anesthesia circuit is intact. Do a quick positive pressure leak test by closing the pop-off valve, occluding the Y-piece and pressurizing the circuit to 30 cm H_2O by pressing the oxygen flush button.
Endotracheal tube		The endotracheal tube should be checked, including the cuff patency.
Syringe		A 5-10ml syringe should be immediately available to inflate the endotracheal tube cuff following intubation.
Stylet		A stylet helps maintain appropriate endotracheal tube tube tip curvature and stiffness during intubation.
Laryngoscope with functional light		A quick check of the light and function of the laryngoscope is important and takes just

		seconds. Backup equipment such as a video laryngoscope should be available.
Functional suction		The suction system (Yankauer suction tip, suction tubing and canister) must be intact and functional to allow you to rapidly clear secretions and body fluids such as vomitus.
Prepares medications		The induction agent and muscle relaxant (succinylcholine) must be prepared quickly.
**Induction/Intubation**		
Pulse oximeter audible		Standard of care is maintenance of the pulse oximeter variable pitch pulse tone and the low threshold alarm at levels that are audible to the anesthesiologist.
Blood pressure monitor cycling		The blood pressure must be cycled frequently throughout the case. Normal maternal blood pressure is important for adequate uteroplacental perfusion.
Electrocardiogram functioning		The electrocardiogram must be verified to be functioning.
Verify obstetric team readiness		To limit the interval between induction and delivery, in order to minimize the risk of neonatal depression, confirm that the obstetric team will be prepared to proceed following induction.
Apply cricoid pressure		Doubts have been raised about the efficacy of cricoid pressure, however it is still widely used. An assistant should be asked to provide firm backward continuous

		pressure on the cricoid cartilage to occlude the esophagus during induction and until the airway has been secured.
Administer induction agent		Induction of anesthesia is typically performed after the abdomen has been prepared and draped. Propofol is the most commonly used agent.
Administer succinylcholine		Succinylcholine is the muscle relaxant of choice for a rapid sequence induction, unless otherwise contraindicated.
Wait for medication effect		Onset of paralysis may be preceded by fasciculations, but may be less prominent in pregnancy.
Direct laryngoscopy		Airway mucosa is more edematous and friable during pregnancy. Caution must be taken during direct laryngoscopy to avoid trauma to tissues and repeated attempts.
Pass endotracheal tube		Pass the styletted endotracheal tube under direct vision into the trachea. A tube diameter no greater than 7.0 mm is typically recommended.
Inflate cuff		Inflation of the endotracheal tube cuff with approximately 4 ml of air usually produces adequate cuff pressure of 20 – 30 cm H_2O.
Confirm presence of end-tidal carbon dioxide		Correct placement of the endotracheal tube must be verified by continuous carbon dioxide analysis of the expired gas.
Notify obstetrician to proceed		In the high pressure circumstance of emergency

		cesarean delivery, clear communication about exactly when you are ready for the obstetricians to make incision is critical. Poor communication could lead to the surgeons making incision prematurely.
Release cricoid pressure		Cricoid pressure should be released once confirmation of correct endotracheal tube placement has occurred.
Confirm bilateral breath sounds		Although not 100% reliable, equal and bilateral breath sounds help to rule out endobronchial intubation.
Secure endotracheal tube		The endotracheal tube must be secured in place with adhesive tape to prevent dislodgement.
**Intraoperative management**		
Before delivery		
Initiate mechanical ventilation		Controlled mechanical ventilation must be initiated after tracheal intubation.
Appropriate tidal volume/respiratory rate		Tidal volume of 6- 8 ml/kg should be provided and the minute ventilation titrated appropriately, based on the end-tidal CO_2. Hyperventilation should be avoided. Severe hypocapnia may impair uteroplacental blood flow due to uterine artery vasoconstriction and shift the oxygen-hemoglobin dissociation curve to the left.
Maintain $FiO_2 \geq 0.5$		Inhaled oxygen of at least 50% should be provided until delivery.
Maintain inhaled agent ≥ 1 MAC		MAC is said to be lower in pregnancy, however cesarean sections with

		general anesthesia are associated with a greater risk of awareness. An adequate depth of anesthesia for maintenance of anesthesia must be quickly achieved. Nitrous oxide is usually administered to supplement the volatile anesthetic.
Protect eyes		Prevent corneal abrasions by taping eyelids closed.
Orogastric tube placed and suctioned		Decompression of the stomach by suctioning via an orogastric tube reduces the volume of stomach contents and may reduce the risk of pulmonary aspiration during emergence.
Esophageal temperature probe placed/temperature monitored		Hypothermia is a significant risk during general anesthesia and temperature monitoring should be performed.
Peripheral nerve stimulator placed		Peripheral nerve stimulator use facilitates objective monitoring of the depth of neuromuscular blockade.
After delivery		
Oxytocin infusion		An oxytocin infusion at 15 units per hour is administered after delivery to facilitate firm contraction of the uterus and minimize bleeding. In the event of uterine atony, this rate is increased to 30 units/hour.
Decrease inhaled agent ≤ 0.5 MAC		The volatile inhaled agent is typically lowered to ≤ 0.5 MAC to limit dose-dependent uterine relaxation.
Administer nitrous oxide		Nitrous oxide 50- 70% is used to supplement the volatile agent.

Administer opioid as needed		Opioid administration is often delayed until after delivery to minimize the risk of neonatal depression.
Administer hypnotic as needed		Midazolam or other hypnotics may be added as needed to increase the depth of anesthesia.
Administer muscle relaxant as needed		Additional muscle relaxant may or may not be needed when the relaxation from succinylcholine has worn off.
