# Supplementary material for: Electronic Feedback Alone Versus Electronic Feedback Plus in-Person Debriefing for a Serious Game Designed to Teach Novice Anesthesiology Residents to Perform General Anesthesia for Cesarean Delivery: Randomized Controlled Trial
Source: JMIR Serious Games. 2024 Nov 19;12:e59047. doi: 10.2196/59047 (PMC11611795; doi:10.2196/59047)
Supplement: Multimedia Appendix 3 [file games-v12-e59047-s003.pdf]

Q1*

What is your age range (years)?

- < or = 25
- 26 - 30
- 31 -35
- 36 - 40
- > or = 41

Q2*

What is your gender?

- Female
- Male
- Not listed. Please specify in the text box.

Q3*

How much experience have you had performing general anesthesia for cesarean delivery ?

- Never
- 1 - 2 times
- 3 - 5 times
- 5 - 10 times
- >11 times

Q4*

How much experience have you had performing general anesthesia in pregnant women for non-obstetric surgery? Examples include, but are not limited to appendectomy, cholecystectomy, or neurosurgery.

- Never
- 1 - 2 times
- 3 - 5 times
- 5 - 10 times
- > 11 times

Q5*

How often do you play video games? This includes any kind of digital game - from puzzle games on your mobile phone, to racing or sports games, and 3-D action games, etc.

- Never
- Rarely (1 time per year or less)
- Occasionally (1 - 6 times per year)
- Often (7 - 12 times per year)
- Very often (> once per month)

Q6*

How often do you play the following types of video games? [**See the question above for the definitions of the frequency**].

	Frequency of playing video games				
	Never	Rarely	Occasionally	Often	Very Often
Puzzles or brain games (e.g. Tetris)					
Sports games (e.g. Madden, NFL 24)					
Educational games (e.g. Anesthesia SimSTAT, HumanSim, Foldit)					
Computer-based simulation (e.g. flight simulator)					
First person shooter 3-D games (e.g. Fortnite, Gran Turismo, Halo 5)					
Other (specify)					

Q7*

How easy was it to use the serious game (on a scale of 1 - 5)?

- 1 = Not at all easy to use
- 2 = Slightly easy to use
- 3 =Somewhat easy to use
- 4 = Quite easy to use
- 5 = Very easy to use

Q8*

Can you give us a reason for your answer above?

Q9*

How would you rate your level of mental effort required to play the serious game
(scale 1 - 9, **1 = very very low mental effort**, **9 = very very high mental effort**)?

	1	2	3	4	5	6	7	8	9
Click to write Choice 1									

Q10*

Can you give us a reason for your answer above?

Q11*

How would you rate the level of stress you felt while playing the game (on a scale of 1 - 5)?

- 1 = I felt no stress at all
- 2 = I felt slightly stressed
- 3 = I felt somewhat stressed
- 4 = I felt quite stressed
- 5 = I felt very stressed

Q12*

Can you give us a reason for your answer above?

Q13*

How would you rate the realism of today's serious game experience (on a scale of 1 - 5)?

- 1 = Not at all realistic
- 2 = Slightly realistic
- 3 = Somewhat realistic
- 4 = Quite realistic
- 5 = Very realistic

Q14*

How satisfied were you with the ***electronic*** feedback/debriefing at the end of the serious game (on a scale of 1 - 5), with respect to helping you learn about managing this scenario?

- 1 = Not at all satisfied
- 2 = Slightly satisfied
- 3 = Somewhat satisfied
- 4 = Quite satisfied
- 5 = Very satisfied

Q15*

If you received **in-person** feedback immediately after you played the serious game the *first time*, how satisfied were you with the in-person feedback (on a scale of 1- 5), with respect to helping you learn to manage this scenario? If you did NOT receive in-person feedback, select the last option - **N/A.**

- 1 = Not at all satisfied
- 2 = Slightly satisfied
- 3 = Somewhat satisfied
- 4 = Quite satisfied
- 5 = Very satisfied
- N/A - I did not receive in-person feedback after playing the game the first time

Q16*

What new information, if any, did you learn from playing the serious game?

Q17*

How much do you agree with the statement, "*Knowledge gained from playing serious video games can be transferred to the clinical setting*" (on a scale of 1 - 5)?

- 1 = Strongly disagree
- 2 = Disagree
- 3 = Neither agree nor disagree
- 4 = Agree
- 5 = Strongly agree
