# Supplementary material for: Electronic Feedback Alone Versus Electronic Feedback Plus in-Person Debriefing for a Serious Game Designed to Teach Novice Anesthesiology Residents to Perform General Anesthesia for Cesarean Delivery: Randomized Controlled Trial
Source: JMIR Serious Games. 2024 Nov 19;12:e59047. doi: 10.2196/59047 (PMC11611795; doi:10.2196/59047)
Supplement: Multimedia Appendix 4 [file games-v12-e59047-s004.pdf]

Survey free text responses regarding participant perceptions of the experience of playing EmergenCSim™

"The tutorial helped a lot and informed where you have to click to have a certain action. It was helpful to see the options you could choose before being in the actual game."

"I think the practice simulation is very helpful. Allows you to get oriented and get what you are looking for".

"Most actions were intuitive but finding a specific action through the user interface was a little confusing at first."

"There were too many clicks to start an action (having to click the patient, click the folder, then click the action, then click the prompt text to actually perform task). This took a long time and there were many instances where I didn't click precisely enough to perform and intended action and thus had to re-click through all those steps."

"If I wanted for example to start the oxygen, I would have to look around the game and try to figure out how to do it."

"For things that I would normally do simultaneously (i.e., checking machine, ensuring presence of airway equipment) I had to constantly remind myself to do in the simulation."

Survey free text responses regarding the level of stress felt while playing EmergenCSim™

"The music at the beginning set the stage."

"Every time the surgeon asked if they could make incision, was stressful."

"The verbalizations from surgeon and patient, as well as monitor beeps added to the stress level (probably appropriately)."

"Situation was stressful, but the simulation being a video game made it more enjoyable and less stressful."

Free text responses to survey question, "What new information, if any, did you learn from playing the serious game?".

Better ways to react to emergency situations
Give antacid
Learned about the sequence for cricoid pressure, as well as sequence for volatile anesthetic management
Critical components of providing safe anesthesia for emergency obstetric cases.
Prioritizing actions for stat section
How volatile agent dosing changes after the baby is delivered, importance of sodium citrate, importance of cricoid pressure and how to give it, doses of propofol, succinylcholine
Fraction of inhaled anesthetics/nitrous during GA for cesarean section
I learned about the value of using sodium citrate and was reminded about the importance of always checking an IV in an emergent situation
It clarified some of the questions I had from an emergent cesarean section that I was unable to find out during my first GA case a few days ago. It also helped confirm the workflow of steps to take in this emergent situation.
Communication with surgery
Steps for rapid sequence induction in pregnancy, induction medications
Give sodium citrate before rapid sequence induction, surgeon can cut after end-tidal CO_2 confirmed
How to manage volatile anesthetic during cesarean section, the order/steps that are important in an emergency
Use of bicitra, gastric emptying, the right sequence and force for cricoid pressure. When to make incision
How to do general anesthesia for cesarean section!
Nitrous oxide application
Bicitra for stat cesarean section
I learned more about obstetric physiology
The flow of a stat cesarean delivery, the indicated (and contraindicated or inappropriate) steps to take in such a situation, and the optimal sequence of actions to prioritize the most time-sensitive actions in an urgent, high-stress format.
Gastric prophylaxis prior to induction of general anesthesia
Dosing of sodium citrate
I haven't been through this scenario in real life so much of this is learning such as the continued reminders from the surgeons that they are ready to get started
I learned it's good to practice without the pressure that would be associated with this if this were the real scenario
Learned and reinforced equipment checks prior to emergency cesarean section as well as focused patient history questions.
None, reinforced previously learned information

I learned about sodium citrate and when to give it, and when to give opioid analgesic.
To not lower your FiO_2 below 50% in a cesarean section, to always administer additional anesthetic besides inhaled agents
Volatile agent use during pregnancy and after delivery
How to give gases before and after delivery
Sequence, most important events, importance of volatile agents
Order of doing things in the operating room in this scenario
Dosages, order of priorities
To tilt the bed 15 degrees to the left to improve venous return
I learned a lot regarding management for cesarean sections
Sodium citrate before induction, giving midazolam/fentanyl after delivery of baby
I learned the sequence of events for rapid sequence induction, learned about saving fentanyl/midazolam until after baby delivered, learned about importance of calling out communication to surgeon actively
It definitely helped to have gone through the game to think about the steps I should do.
Taking note of important steps involved in emergent patient care and teamwork required to achieve optimal outcomes
How to prioritize things in this urgent scenario
Calling for help early is critical, and it is easy to forget priorities in a chaotic situation
Pre-medication with antacids
General management of general anesthesia for stat obstetric cases, Practical and academic considerations
I learned about the splash preparation of the abdomen occurring before we intubate and bicitra being near instantaneous effect
Some of the critical vs. non-critical steps, timing of certain steps
Intraoperative sequence of events.
Learned a lot about intra-operative management of emergent cesarean section under general anesthesia, especially since I have never done one.
Giving citrate/fentanyl/midazolam as ?standard for emergent cesarean section. Rapid nature of case.
Communication with the obstetric team, gastrointestinal prophylaxis, timing of narcotics
The workflow of performing tasks under the stressful environment
General maternal physiology and workflow in the operating room for stat cesarean section
Nitrous oxide use
How to proceed in emergent situations
